# Folic Acid-Decorated Lipidic Nanocapsules Co-Loaded with Atorvastatin and Curcumin to Enhance Glioma Targeting in Mice

**DOI:** 10.3390/ph18111623

**Published:** 2025-10-27

**Authors:** Mahitab Bayoumi, John Youshia, O. A. El-Kawy, Sara A. Abdel Gaber, Mona G. Arafa, Maha Nasr, Omaima A. Sammour

**Affiliations:** 1 Department of Pharmaceutics and Pharmaceutical Technology, Faculty of Pharmacy, The British University in Egypt, Cairo 11837, Egypt; 2 Department of Pharmaceutics and Industrial Pharmacy, Faculty of Pharmacy, Ain Shams University, Cairo 11566, Egypt; 3 Labelled Compounds Department, Egyptian Atomic Energy Authority, Cairo 13759, Egypt; 4 Department of Biochemical Engineering, Faculty of Energy and Environmental Engineering, The British University in Egypt, Cairo 11837, Egypt; 5 Nanomedicine Department, Institute of Nanoscience and Nanotechnology, Kafr Elsheikh University, Kafr Elsheikh 33516, Egypt; 6 Chemotherapeutic Unit, Mansoura University Hospitals, Mansoura 35516, Egypt; 7 Nanotechnology Research Center, The British University in Egypt, Cairo 11837, Egypt; 8 Department of Pharmacology and Therapeutics, College of Medicine and Health Sciences, United Arab Emirates University, Al Ain 15551, United Arab Emirates

**Keywords:** glioma, lipidic nanocapsules, drug delivery, radiolabeling, atorvastatin, curcumin

## Abstract

**Background**: Glioma remains an intractable and highly aggressive brain tumor, mainly due to the daunting obstacle presented by the blood–brain barrier (BBB). To overcome this challenge and enhance therapeutic efficacy, a dual-drug delivery system was engineered. This system co-encapsulated curcumin, a nutraceutical with multitargeted anticancer potential, with atorvastatin calcium, a repurposed anticancer agent, within lipidic nanocapsules (LNCs). **Methods**: LNCs were prepared via the phase inversion temperature method and optimized using a Box–Behnken design. The optimized LNCs were subsequently functionalized with folic acid (FA) to enable active targeting. FA-LNCs were characterized using XPS, TEM, in vitro release, and MTT cytotoxicity assays. Atorvastatin and curcumin were radiolabeled separately with iodine-131 to evaluate the in vivo pharmacokinetics in a glioma-bearing mouse model. **Results**: The optimized LNCs and FA-LNCs displayed a mean particle size of 97.98 ± 2.27 nm and 181.60 ± 2.83 nm, a polydispersity index of 0.32 ± 0.07 and 0.40 ± 0.02, and a zeta potential of −15.85 ± 1.35 mV and −11.90 ± 2.80, respectively. XPS and FTIR analyses verified FA conjugation. Both LNCs and FA-LNCs enhanced the in vitro cytotoxicity compared to free drugs; however, the most pronounced effect of FA functionalization was observed in vivo. Most significantly, FA-LNCs achieved markedly greater glioma accumulation than non-functionalized LNCs, with AUC values 2.0-fold higher for atorvastatin and 2.6-fold higher for curcumin. When compared to the free drug solutions, this efficiency was even more pronounced, with atorvastatin and curcumin showing enhancements of 8.2 and 12.4 times, respectively. **Conclusions**: FA-LNCs markedly improved glioma targeting efficiency and reduced systemic clearance, which underscores the therapeutic potential of integrating nutraceuticals with repurposed agents to achieve effective glioma therapy.

## 1. Introduction

Gliomas, especially glioblastoma multiforme, are among the most lethal primary brain tumors, affecting approximately 3 out of 100,000 people each year [1]. The 5-year survival rate is less than 10%, and the median survival is still only 14–17 months even with aggressive treatments like radiotherapy, temozolomide chemotherapy, and maximal safe surgical resection [2]. Tumor invasiveness, blood–brain barrier-imposed drug delivery hurdle, and tumor heterogeneity all restrict the efficacy of treatment and increase resistance and recurrence [3]. Thus, there is an urgent need for innovative therapeutic approaches that can overcome obstacles and significantly improve patient outcomes.

Drug repositioning and nutraceutical-based strategies present promising avenues for cancer therapy by repurposing agents with established safety profiles and utilizing bioactive natural compounds with multitargeted effects [4,5]. Nutraceuticals like curcumin and nobiletin have chemosensitizing, anti-inflammatory, and antioxidant effects, while repositioned drugs like statins and antidiabetics have shown anticancer activity by altering important survival pathways. In the context of glioma, atorvastatin has been recently repositioned as a promising candidate with confirmed efficacy alone or in combination with conventional therapies. Studies suggest that atorvastatin exert anti-glioma activity through multiple pathways, including induction of apoptosis (via caspase-8–caspase-3 activation and Bcl-2 suppression), inhibition of invasion and migration (via downregulation of MT1-MMP, MMP-2, and MMP-9), suppression of MAPK and JNK signaling, and promotion of autophagy—with potential synergistic effects when combined with temozolomide [6,7,8,9,10,11,12]. Curcumin exerts its anti-glioma effects through multiple molecular mechanisms, including suppression of MMPs, ROS-mediated apoptosis, inhibition of SHH/GLI1 and STAT3 signaling, and modulation of NF-κB, PI3K/Akt, JAK/STAT, and MAPK pathways, ultimately leading to cell cycle arrest, apoptosis, and reduced invasion and [13,14,15,16,17,18]. Together, the complementary mechanisms of curcumin and atorvastatin demonstrate their potential as a synergistic drug combination, offering compelling justification for their co-encapsulation in nanocarriers to improve therapeutic efficacy against gliomas.

Nanotechnology is a promising strategy to deliver drugs to the brain; however, nanocarriers must typically have particle sizes below 200 nm to efficiently deliver drugs across the blood–brain barrier (BBB). This is because smaller particles allow transcytosis and prolong systemic circulation while avoiding rapid clearance by the mononuclear phagocyte system (MPS) [19]. Beyond particle size, certain physicochemical characteristics are frequently linked to effective drug molecule brain penetration. According to recent research, substances with molecular weights typically between 400 and 500 Da, few hydrogen bond donors and acceptors, moderate lipophilicity (ideally logP between 1.5 and 2.5) and low plasma protein binding are more likely to cross the BBB [20,21]. Lipidic nanocapsules (LNCs) represent a promising family of nanocarriers for drug delivery, offering a unique combination of the stability associated with lipid-based nanoparticles and the versatility of polymeric carriers. LNCs are composed of a hydrophobic core formed of oils (10–25% *w*/*w*). This hydrophobic core is surrounded by a surfactant shell (10–40% *w*/*w*) like solutol HS15 (a derivative of polyethylene glycol) and lecithin (1–1.5% *w*/*w*), which spontaneously assembles at the oil–water interface into a bilayer structure. Besides enhancing colloidal stability, these surfactants enhance biocompatibility and reduce uptake by the MPS. The aqueous phase, typically a salt solution (1–5% NaCl), facilitates spontaneous LNCs formation via the phase inversion temperature (PIT) method [22,23,24]. LNCs are a promising candidate for glioma treatment. Their small particle size enables effective crossing of the blood–brain barrier, and they can also be easily functionalized to target specific tumor cells, thereby increasing drug delivery efficiency and minimizing side effects. Additionally, their biodegradable nature ensures safe elimination from the body. Their ability to encapsulate various therapeutic agents, including chemotherapy drugs and gene therapies, makes LNCs a versatile platform for the development of novel treatments against glioblastoma [25]. LNCs entrapping various drugs such as sorafenib [26], methotrexate [27], gemcitabine [28], paclitaxel [29], baicalin [30], curcumin [18] and simvastatin [31] showed efficacy in treatment of glioma.

Functionalizing nanocapsules with targeted ligands, such as folic acid (FA), is considered a pivotal strategy for improving drug delivery to the brain. FA is known as a tumor-associated antigen, since folate receptor expression levels on tumor cell surfaces are 100 to 300 times higher than in normal cells. This differential expression facilitates receptor-mediated endocytosis, allowing for preferential nanoparticle uptake by malignant cells, thereby enhancing drug accumulation within the tumor while minimizing off-target effects [32,33,34]. FA functionalization has been shown in many studies to significantly improve brain penetration and glioma targeting specifically via receptor-mediated endocytosis, and achieve higher tumor uptake than with non-targeted delivery systems [35,36,37].

In order to monitor the nanocarrier fate in vivo, radiolabeling is considered a gold standard because it provides better sensitivity and quantitative precision than conventional analytical methods. It helps to understand how nanocarrier design affects medication distribution and pharmacokinetics, which is particularly helpful in the context of glioma therapy. Previous studies have successfully evaluated brain-targeted formulations and tumor localization using iodine-131 (^131^I) radiolabeling [38,39,40]. This idea is furthered in the current work by using a sequential radiolabeling technique. This strategy permits independent pharmacokinetic profiling of distinct payloads by labeling each drug in a co-loaded system in turn, hence offering new insights into the behavior of dual-drug nanocarriers. Tumor induction was carried out by percutaneous induction of CT-2A murine glioma cell. The CT-2A model is a well-characterized syngeneic, immunocompetent model that captures many important characteristics of human high-grade gliomas, including high cellular density, invasiveness and glioma stem cell (GSC) populations [41,42].

According to previous studies, peptide- or folic acid-modified nanoconstructs have demonstrated improved site-specific targeting, reduced clearance, and resulted in effective glioma growth inhibition in preclinical models [43]. Therefore, the current study focused on co-encapsulating curcumin and atorvastatin calcium into LNCs, then decorating the surface with FA to improve tumor targeting. The potential of the nanocarriers in glioma therapy was assessed by determining their physicochemical properties and surface functionalization. In addition, the biodistribution and pharmacokinetics of the developed nanocarriers were evaluated in glioma-bearing mice to assess their potential for targeted brain delivery.

## 2. Results

### 2.1. Determination of Particle Size (PS), Polydispersity Index (PDI) and Zeta Potential (ZP) of Atorvastatin and Curcumin-Loaded Lipidic Nanocapsules (At-Cu LNCs)

**Figure 1:**
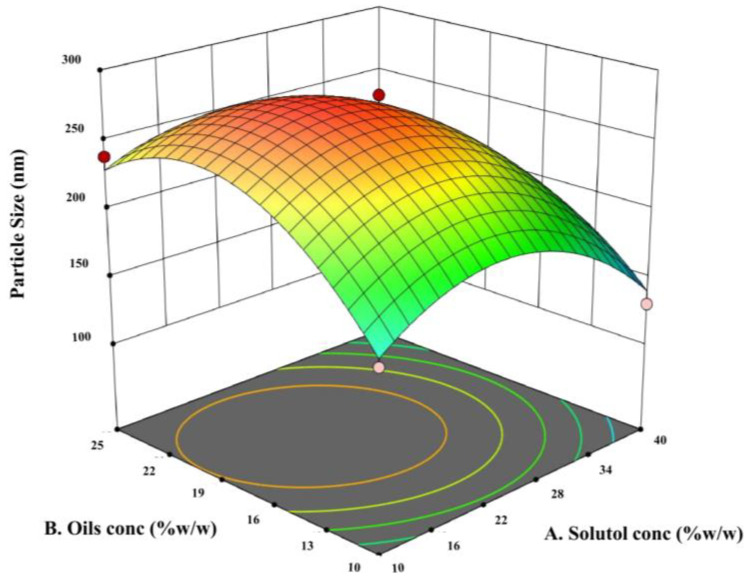
Response surface graph for the effect of different variables on the PS of LNCs formulations.

PS values ranged from 120.75 ± 14.07 nm to 282.55 ± 8.27 nm and PDI values were less than 0.5 in most formulations, reflecting overall moderate narrow size distribution. As illustrated in Table 1, Table 2 and Table 3 and Figure 1, statistical analysis using Design Expert^®^ software version 13 confirmed the significance of the developed model for predicting the particle size of the At-Cu LNCs. The quadratic model for particle size exhibited high significance (*p* ≤ 0.001) and excellent predictive power (R^2^ = 0.9810; adjusted R^2^ = 0.9467), with solutol concentration (A), oil concentration (B), and their quadratic terms (A^2^, B^2^), along with the quadratic term of drug amount (C^2^), contributing significantly to the response. The models showed non-significant lack of fit, indicating good agreement between experimental and predicted values.

The final quadratic equation generated by Design Expert^®^ software for predicting the particle size of At-Cu LNCs, in terms of actual formulation variables, is expressed as follows:

Particle Size = −881.80069 + 10.82292 (Solutol conc) + 39.01056 (Oils conc) + 58.67861× (Amount of drugs) − 0.102222 (Solutol conc × Oils conc) − 0.004778 (Solutol conc × Amount of drugs) − 0.084000 (Oils conc × Amount of drugs) − 0.212639 (Solutol conc^2^) − 0.915667 (Oils conc^2^) − 1.23589 (Amount of drugs^2^)

Increasing solutol concentration resulted in a significant reduction in particle size (*p* ≤ 0.05), as observed in multiple formulation pairs (At-Cu LNCs3 vs. LNCs4, LNCs5 vs. LNCs6, and LNCs7 vs. LNCs8), consistent with previous reports [44,45]. Most formulations exhibited good size distribution uniformity (PDI < 0.5) with statistically insignificant differences among different formulations (*p* > 0.05), which was considered acceptable for nanocarrier systems [46].

Conversely, increasing oil concentration led to a significant enlargement in PS (*p* ≤ 0.05) in formulations with intermediate drug levels (At-Cu LNCs2 vs. LNCs4 and LNCs1 vs. LNCs3) and insignificant enlargement in PS (*p* > 0.05) in formulations of low (15 mg) or high (30 mg) drug levels (At-Cu LNCs9 vs. LNCs11 and LNCs10 vs. LNCs12), attributed to expansion of the lipid core, as supported by previous studies [47,48,49]. Interestingly, variations in drug concentration alone did not significantly impact PS (*p* > 0.05), aligning with earlier findings [50,51]. All LNCs exhibited negative surface charges, and the absolute value of the ZP decreased significantly (*p* ≤ 0.05) as the solutol content increased.

Table 3 presents the optimized formulation composition as selected by Design-Expert^®^ software, with the predicted and experimentally determined results. The results indicate no significant difference between the predicted and measured values (*p* > 0.05), which reflects the accuracy and reliability of the optimization model. It is worthy to note that the PDI of the optimized formulation was 0.32 ± 0.07, and its zeta potential was −15.85 ± 1.35 mV.

### 2.2. Determination of PS, PDI and ZP of Folic Acid Functionalized Nanocapsules (FA-At-Cu LNCs)

PS, PDI and ZP of the FA-At-Cu LNCs containing 10, 20, and 30 mg of FA were analyzed as presented in Table 4. Notably, there was considerable precipitation when 40 mg of FA was added, which indicates poor solubility at this level. Therefore, 30 mg FA was selected as the best quantity, which represents the highest level of FA capable of being solubilized efficiently in the system with optimal physicochemical characteristics. As the amount of FA increased, the PS of the LNCs increased significantly (*p* < 0.05). FTIR data revealed the complete disappearance of the characteristic free carboxylic acid peak of FA at 2500–3500 cm^−1^ in the FA-At-Cu LNC spectrum, as well as the absence of the broad O–H stretching band of Solutol at 3371.71 cm^−1^ from the FTIR spectrum of FA-At-Cu LNCs (Supplementary Figure S1).

### 2.3. X-Ray Photoelectron Spectroscopy (XPS) for Optimized At-Cu LNCs and FA-At-Cu LNCs

**Figure 2:**
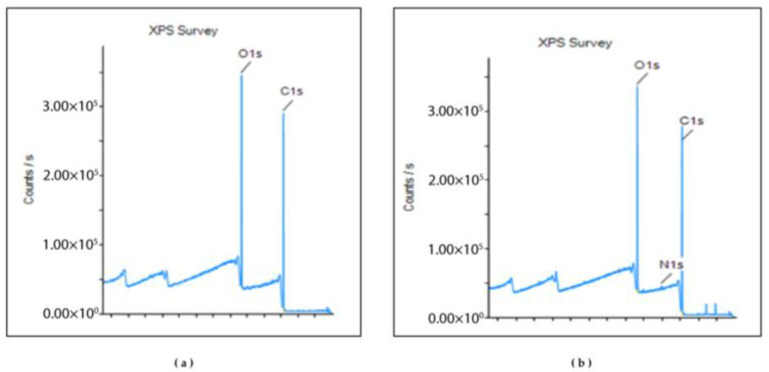
XPS analysis of (**a**) At-Cu LNCs and (**b**) FA- At-Cu LNCs, displaying the nitrogen peak in the latter.

The XPS spectra of At-Cu LNCs and FA-At-Cu LNCs are shown in Figure 2, indicating the presence of nitrogen in the functionalized LNCs attributed to the FA.

### 2.4. Morphology by Transmission Electron Microscopy (TEM) for Optimized At-Cu LNCs and FA-At-Cu Lncs

**Figure 3:**
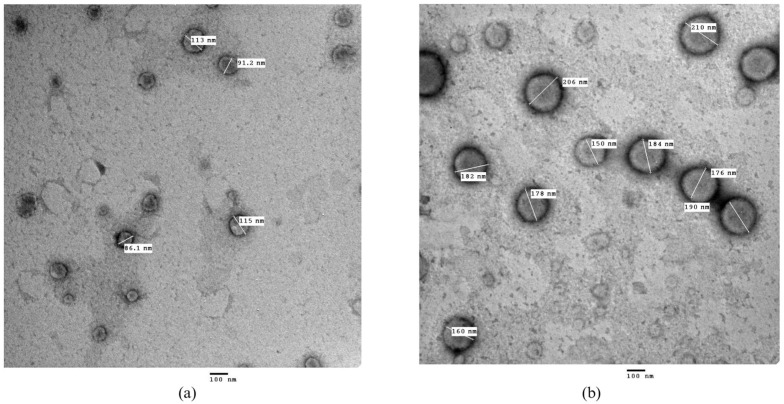
Negative stain electron micrographs of: (**a**) At-Cu LNCs, (**b**) FA-At-Cu LNCs.

As evident in Figure 3, the nanocapsules displayed spherical, non-aggregated morphology with a narrow size distribution, comparable to results reported by other researchers [52,53]. The PS obtained using the TEM imaging concurred with the PS results obtained by the particle size analyzer for At-Cu LNCs (97.98 ± 2.27 nm) and for the FA-At-Cu LNCs (181.60 ± 2.83 nm).

### 2.5. In Vitro Drugs’ Release Evaluation

**Figure 4:**
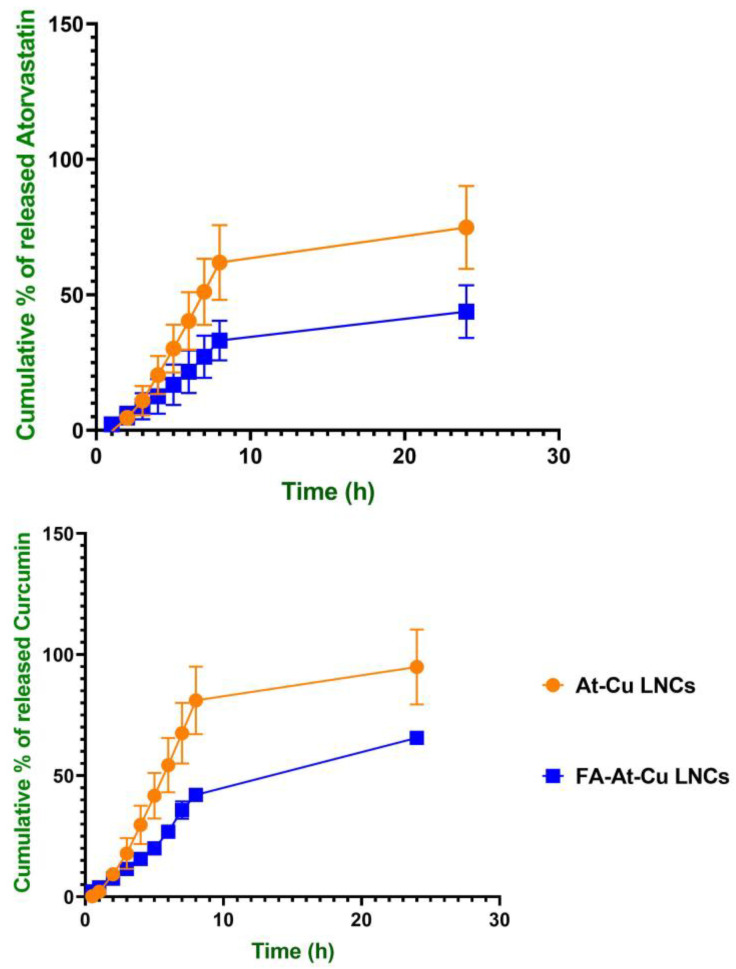
Cumulative percent of atorvastatin and curcumin released from At-Cu LNCs and FA-At-Cu LNCs in deionized water (pH of 7.4 and 4% Kolliphor RH 40) at 37 ± 2 °C over a period of 24 h.

As evident in Figure 4, after a 24 h period, the optimized At-Cu LNCs demonstrated a release of 74.89 ± 15.27% for atorvastatin and 94.89 ± 15.47% for curcumin, which were insignificantly different (*p* > 0.05). Notably, the incorporation of FA onto the LNCs resulted in a substantial reduction in the release of both drugs. Specifically, the release of atorvastatin decreased to 43.83 ± 9.68%, while curcumin release was 65.61 ± 1.95%.

### 2.6. The Effect of Storage on the Physical Stability of the Selected LNCs

Stability studies assessed the physicochemical properties of At-Cu LNCs and FA- At-Cu LNCs over 90 days at 4 ± 1 °C. Specifically, changes in PS, PDI and ZP were monitored. Table 5 summarizes the impact of storage on these parameters. Both formulations exhibited good physical stability, demonstrating overall statistically insignificant changes (*p* > 0.05) in PS, PDI, and ZP throughout the study period.

### 2.7. MTT Assay for Cell Viability

**Figure 5:**
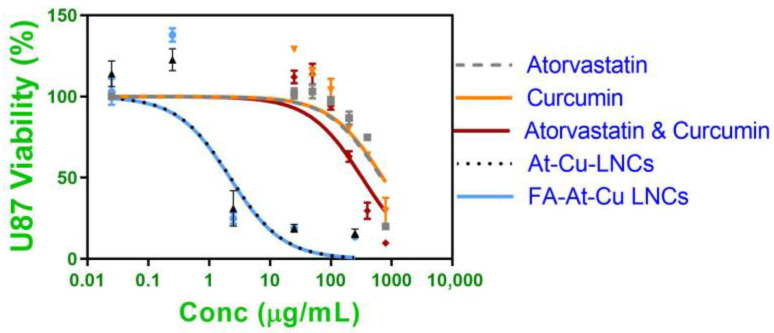
Viability of U87 cells treated for 24 h with At-Cu LNCs (0–250 µg/mL), FA-At-Cu LNCs (0–250 µg/mL), Free atorvastatin solution (0–800 µg/mL), Free curcumin solution (0–800 µg/mL) and Free atorvastatin & curcumin solution (0–800 µg/mL). Symbols represent mean values ± S.D. of cell viability at each concentration, and lines represent non-linear regression fitting of the dose response curve.

The cell viability results clearly demonstrated a dose-dependent cytotoxic effect across all tested formulations, and the IC_50_ values highlighted significant differences between the free drug solutions and the nanoformulations, as shown in Figure 5. Both atorvastatin and curcumin showed limited cytotoxicity to glioma cells when applied individually, with high IC_50_ values of 679.70 ± 17.17 and 723.80 ± 78.35 µg/mL, respectively. Co-administration of the two drugs demonstrated a synergistic effect, reducing the IC_50_ to 328.87 ± 87 µg/mL. However, a substantial enhancement in cytotoxic efficacy was achieved upon encapsulation into LNCs. At-Cu LNCs displayed a dramatically lower IC_50_ of 2.35 ± 0.81 µg/mL, while FA-At-Cu LNCs showed an IC_50_ of 2.21 ± 0.28 µg/mL. MTT cytotoxicity assay findings underscore the superior performance of the nanocarrier system in enhancing cellular uptake and cytotoxic response. Worth mentioning that the difference between At-Cu FA-LNCs and At-Cu LNCs cytotoxicity was insignificant (*p* > 0.05).

### 2.8. In Vivo Study

#### 2.8.1. Radiolabeling Techniques of Atorvastatin, Curcumin, Optimization and Validation

Complementary analytical techniques were used to verify the radiochemical purity of the radiolabeled drugs. Regarding [^131^I] atorvastatin, HPLC verified purity with retention times of 8.50 ± 0.30 min and 1.10 ± 0.20 min, respectively, while thin layer chromatography demonstrated clear separation with Rf values of 0.4–0.5 for the labeled drug and 0.9 for free iodide. Likewise, [^131^I] curcumin was confirmed by paper electrophoresis and RP-HPLC, where radiolabeled curcumin stayed at the origin while free iodide moved towards the anode. Curcumin was identified by HPLC, and its radiolabeled form eluted in 10–12 min, showing free iodide as a clear peak at ~2 min.

Radiolabeling conditions for iodine-131-labeled atorvastatin and curcumin were systematically optimized by evaluating the effects of pH, substrate concentration, reaction time, and oxidizing agent. For atorvastatin, optimal labeling efficiency (97.30 ± 1.50%) was achieved at pH 7 using 15 µg of drug and a 20 min reaction time with iodogen. For curcumin, the best conditions were pH 7, 20 µg of drug, a 10 min reaction time, and 25 µg of chloramine-T, resulting in 82.50% labeling efficiency (pre-purification). After HPLC purification, radiolabeled curcumin demonstrated a radiochemical purity greater than 98.70%, confirming the suitability of both labeled compounds for subsequent biological evaluation (Appendix A).

#### 2.8.2. Pharmacokinetic Analysis of Atorvastatin and Curcumin in Blood, Brain and Glioma Tissues

**Figure 6:**
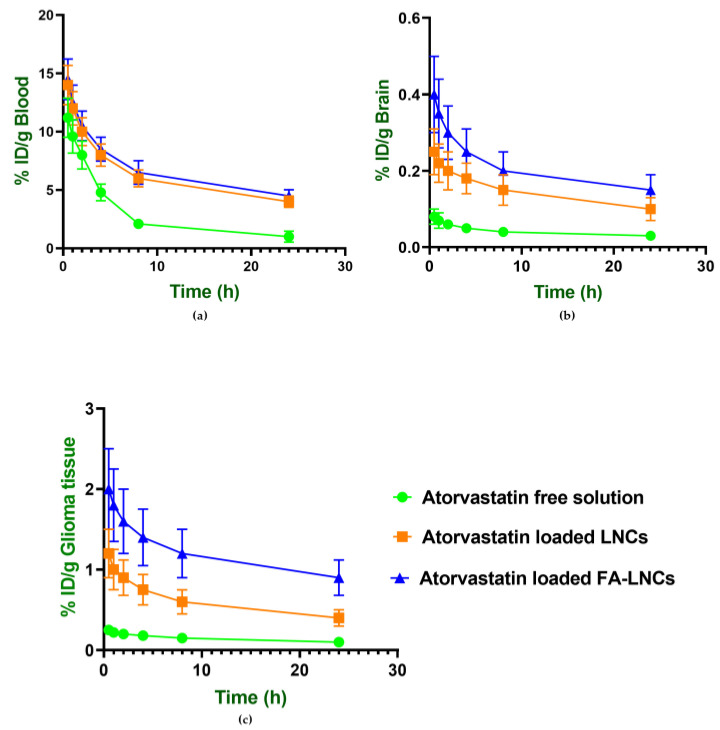
Biodistribution profiles of atorvastatin in blood, brain, and glioma tissue over 24 h following intravenous administration of atorvastatin in free solution and lipidic nanocapsules in glioma-bearing mice in: (**a**) Blood (**b**) Brain and (**c**) Glioma tissue. Data are expressed as % injected dose per gram of tissue (% ID/g).

**Figure 7:**
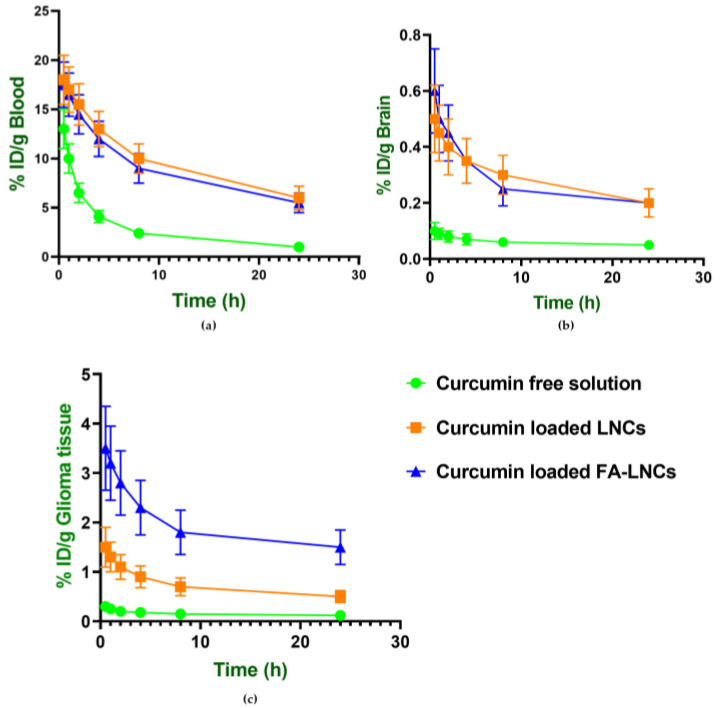
Biodistribution profiles of curcumin in blood, brain, and glioma tissue over 24 h following intravenous administration of curcumin in free solution and lipidic nanocapsules in glioma-bearing mice in: (**a**) Blood, (**b**) Brain and (**c**) Glioma tissue. Data are expressed as % injected dose per gram of tissue (% ID/g).

Pharmacokinetic profiling of atorvastatin and curcumin following intravenous administration of free drug solutions and nanoformulations over 24 h revealed prolonged circulation time and higher tumor accumulation with the nanoencapsulated systems. The mean residence time (MRT) of atorvastatin increased significantly from 9.77 ± 2.57 h (free drug) to over 30 h with nanocarriers, while curcumin MRT rose from 12.08 ± 0.02 h to more than 24 h. Detailed pharmacokinetic parameters and tumor-targeting indices are presented in Table 6, and biodistribution profiles in blood, brain, and glioma tissue are shown in Figure 6 and Figure 7.

When curcumin was used with FA-LNCs, its tumor targeting efficiency increased significantly (*p* < 0.05) from 7.28 ± 0.99% (LNCs) to 20.95 ± 2.51% (FA-LNCs), and its tumor tissue specificity index increased significantly (*p* < 0.05) from 2.51 ± 0.04 (LNCs) to 6.91 ± 0.01 (FA-LNCs). Atorvastatin’s specificity index improved significantly (*p* < 0.05) from 4.15 ± 0.08 (LNCs) to 5.77 ± 0.02 (FA-LNCs), and its targeting efficiency rose significantly (*p* < 0.05) from 9.45 ± 1.79% (LNCs) to a maximum of 17.32 ± 2.89% using FA-LNCs.

#### 2.8.3. Biodistribution in Clearance Organs: Liver, Spleen, Kidneys, and Intestine

**Figure 8:**
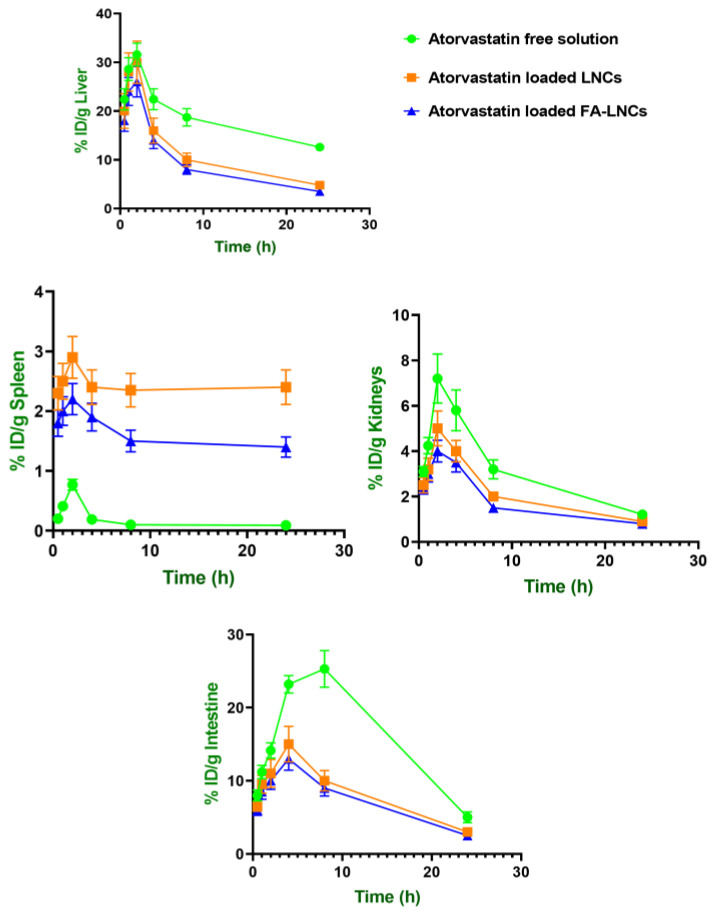
Biodistribution of atorvastatin in clearance-related organs (%ID/g tissue) following intravenous administration of different formulations in glioma-bearing mice.

**Figure 9:**
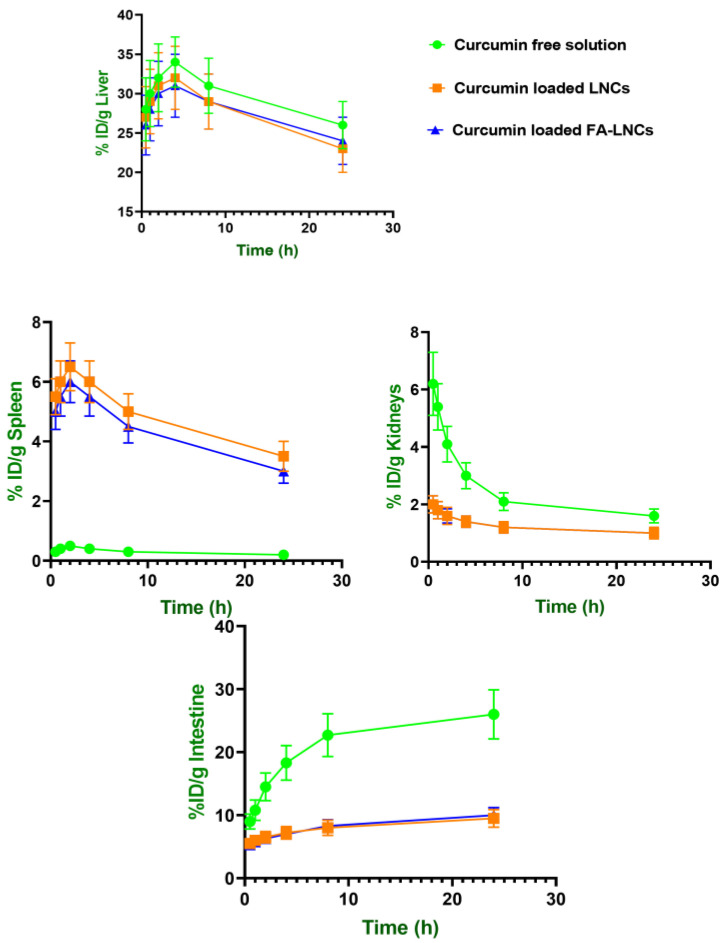
Biodistribution of curcumin in clearance-related organs (%ID/g tissue) following intravenous administration of different formulations in glioma-bearing mice.

The clearance profiles of both atorvastatin and curcumin revealed formulation-dependent differences in accumulation within key elimination organs (liver, spleen, kidneys, and intestine). As evident in Figure 8 and Figure 9 and Table 7, for free atorvastatin solution, the highest AUC values were observed in liver (440.88 ± 49.33 %ID*h/g tissue) and intestine (398.22 ± 52.90 %ID*h/g tissue), with moderate accumulation in kidneys (75.39 ± 14.24 %ID*h/g tissue) and minimal presence in the spleen (3.90 ± 0.90 %ID*h/g tissue). For free curcumin solution, the highest AUC values were similarly recorded in the liver (711.50 ± 114.83 %ID*h/g tissue) and intestine (526.50 ± 111.63 %ID*h/g tissue).

For curcumin in particular, lipidic nanocapsules markedly reduced hepatic clearance compared to the free drug solution, whereas splenic clearance was increased for both drugs. Additionally, both drugs showed reduced renal and intestinal AUC values when encapsulated. Curcumin’s kidney AUC decreased significantly (*p* < 0.05) from 57.87 ± 12.47 to 29.50 ± 6.53 %ID*h/g tissue, and atorvastatin’s from 75.39 ± 14.24 to 50.98 ± 8.84 and 41.95 ± 7.16 %ID*h/g tissue for LNCs and FA-LNCs, respectively, further confirming a shift in elimination from renal pathways to MPS-dominated mechanisms.

#### 2.8.4. Tissue Distribution in Cardiopulmonary, Musculoskeletal, and Endocrine Organs

Minor quantities of radiolabeled atorvastatin and curcumin were detected in the cardiopulmonary, musculoskeletal, and endocrine organs, with levels markedly lower than those observed in the previously discussed organs such as the liver, brain, and glioma (Appendix B).

The systemic safety of the developed nanocapsules was supported by the tiny levels of radioactivity in thyroid gland. In addition, the body weight of mice was tracked throughout the in vivo pharmacokinetic investigations and stayed constant across all groups. Furthermore, the results of the hemolysis assay demonstrated negligible hemolytic activity (<5%).

## 3. Discussion

At-Cu LNCs were prepared by PIT [54]. Lecithin was selected as the lipophilic surfactant of choice to enhance the stability of the LNCs by forming a rigid shell upon cooling. Additionally, it improves compatibility with biological membranes [23,44]. Solutol is composed of PEG 660 and PEG 660 hydroxystearate and performs a critical role in the formation and stabilization of LNCs. It is the most frequently used surfactant in the preparation of LNCs, which also inhibits P-gp drug efflux and consequently causes a significant level of intracellular drug accumulation, which improves the cytotoxicity of the medication on glioma cells in particular [55]. Its hydrophilic–lipophilic balance is temperature-dependent. This allows the PIT to be used, taking advantage of solutol’s ability to adjust its affinity for water and oil based on temperature changes [56]. NaCl lowers solutol’s phase-inversion temperature, enabling LNC production at lower temperatures [50]. The statistical findings obtained by Design Expert^®^ were corroborated by formulation-specific comparisons. The lowest PDI values were mostly observed in formulations with either higher surfactant content or intermediate oil concentrations, highlighting the role of solutol HS15 in stabilizing the interface and minimizing size heterogeneity [57]. This effect is likely due to the amphiphilic nature of solutol, whose PEG moieties and stearate chains reduce interfacial tension and promote stability through lateral interactions [49,58]. All LNCs exhibited negative surface charges, primarily due to lecithin, a zwitterionic phospholipid containing both phosphate and amino functional groups, as well as the surface orientation of the dipolar PEG chains in solutol. ZP decreased as solutol content increased because solutol HS 15 is a non-ionic surfactant made of fatty acid esters and PEG. The PEG chains create a hydrophilic, electrically neutral steric layer that covers the charged groups on the nanoparticle surface, reducing the measured zeta potential. Importantly, this decrease is not necessarily unfavorable, as the steric stabilization imparted by the PEG chains effectively inhibits nanoparticle aggregation even when electrostatic repulsion is reduced [56,59], which was evident in the results of the three months’ stability study, where no aggregation was found, despite the low zeta potential values.

The XPS spectra confirmed the successful conjugation of FA onto the surface of the LNCs, as evidenced by the appearance of a characteristic nitrogen peak. This finding was further supported by FTIR analysis, which revealed the characteristic absorption bands corresponding to folic acid, confirming its successful attachment to At-Cu LNCs surface (Supplementary Figure S1). TEM imaging revealed spherical nanocapsules with sizes consistent with those obtained from dynamic light scattering, supporting the reliability of these measurements.

The observed reduction in drug release from FA-At-Cu LNCs within 24 h, compared to unmodified counterparts, may be attributed to multiple interrelated factors. Firstly, surface functionalization with FA enhances the interfacial stability of the nanocapsule through improved steric and electrostatic stabilization, which reinforces the structural integrity of the lipid shell and limits drug diffusion [60,61,62]. Additionally, at physiological pH (7.4), FA remains largely ionized, contributing to a hydrophilic surface layer that hinders the outward migration of lipophilic compounds such as curcumin and atorvastatin. Several studies have highlighted the pH-dependent drug release characteristics of nanocarriers modified with FA, underscoring the promise of such delivery systems in the context of cancer treatment [63,64,65].

The MTT assay confirmed that encapsulation markedly enhanced the cytotoxicity of curcumin and atorvastatin compared to their free forms. This enhancement can be attributed to efficient endocytosis facilitated by the nanoscale carrier size, the sustained release behavior of the nanocapsules which ensures continuous intracellular exposure, and synchronized intracellular delivery achieved through co-loading, which likely augmented their complementary cytotoxic effects [18,52]. Active targeting strategies might not always lead to a noticeable increase in cellular uptake or cell-killing ability in simple in vitro models. However, their main benefit is their ability to handle the complex biological barriers found in vivo. Unlike static cell cultures, where nanoparticles can easily reach cells, the in vivo environment poses tough challenges. These include quick removal by the MPS, random interactions with unintended cells, and barriers in tissues, such as the blood–brain barrier in glioma. Therefore, active targeting mainly aims to boost the concentration and retention of drugs at the site of disease by overcoming these complex physiological challenges rather than just increasing the rate of receptor uptake in a controlled in vitro setting. Previous reports demonstrated that LNCs exhibit negligible or acceptable cytotoxicity at moderate concentrations, thus supporting their safety as delivery systems [34,52,66,67,68].

LNCs led to a marked increase in brain and glioma bioavailability, supporting the superior glioma-targeting ability of folate-conjugated nanocapsules benefiting from enhanced permeability and retention (EPR) effect, receptor-mediated uptake via folate receptors, and prolonged circulation times. In comparison to their free solutions, curcumin and atorvastatin both showed markedly improved tumor targeting efficiency and specificity when administered via modified nanocapsules. Importantly, FA-LNCs produced pronounced targeting efficiency and specificity indices for both medications, greatly surpassing non-targeted LNCs. This marked superiority of the FA-LNCs underscores the substantial contribution of folate receptor-mediated uptake in achieving highly efficient and specific glioma targeting for both therapeutic agents [37,69].

The clearance profile of atorvastatin aligns precisely with the established pharmacokinetics of the drug: free atorvastatin undergoes rapid organic anion transporting polypeptide 1B1/1B3 (OATP1B1/1B3)-mediated uptake into hepatocytes, where it undergoes extensive CYP3A4 metabolism, followed by predominant elimination through biliary excretion into the feces. Renal excretion of atorvastatin and its active metabolites is known to be a minor pathway, typically accounting for less than 2% of the dose [70,71]. The low splenic AUC further confirms that free, small-molecule drugs do not typically accumulate significantly in MPS organs. For free curcumin solution, the highest AUC values were similarly recorded in the liver and intestine, reflecting its extensive hepatobiliary metabolism and rapid intestinal clearance. Free curcumin binds to plasma proteins (primarily albumin), forming a complex that limits extrahepatic distribution but facilitates passive diffusion into hepatocytes. In hepatocytes, it undergoes rapid glucuronidation/sulfation by UGT/SULT enzymes, generating inactive metabolites excreted into bile. This process results in >90% fecal elimination within 2 h and low tissue accumulation [72,73,74]. Some authors highlighted that the extremely fast elimination kinetics of free curcumin compel that for accurate measurement of plasma concentration, measurements should be performed within the first 5 min post-injection [75]. The kidney AUC was moderately high, consistent with minor renal elimination. In contrast, splenic accumulation remained minimal, confirming the limited uptake of free curcumin MPS organs. These findings align with curcumin’s known pharmacokinetic limitations, including low aqueous solubility, rapid plasma clearance, and substantial hepatic metabolism, which collectively limit its systemic persistence and therapeutic potential without formulation enhancement [76,77,78]. The clearance pathways for curcumin and atorvastatin were markedly changed by nanoencapsulation. Because of the increased uptake by MPS, splenic clearance dramatically increased while hepatic clearance decreased. Although hepatic accumulation of curcumin was reduced by nanoencapsulation, a substantial fraction remained in the liver. However, curcumin is generally regarded as safe, with clinical studies demonstrating a favorable hepatic safety profile even at high doses [79,80]. Splenic macrophages actively sequestering nanocarriers is consistent with the increased splenic deposition [81]. Additionally, after encapsulation, both medications showed decreased renal AUC values, which can be attributed to the size-exclusion effect of nanoparticles that limits glomerular filtration. This confirms a shift in elimination from renal excretion to passage through MPS [82,83]. All formulations showed minimal thyroid uptake (0.03 %ID/g), indicating minimal deiodination and high in vivo stability of the iodine label [84,85]. Good hemocompatibility and tolerability of the formulations were also obtained, further reinforcing their suitability for intravenous administration [86]. Studies on urinary excretion revealed that while free drugs were quickly removed by the kidneys, LNCs dramatically decreased urine clearance, which is in line with the protective effect of nanoencapsulation and decreased glomerular filtration [87,88]. It is worth mentioning that complementary in vitro stability assessments confirmed that the radiolabeled atorvastatin and curcumin maintained radiochemical integrity (>95%) under both physicochemical and serum incubation conditions for the whole study duration, supporting the reliability of the pharmacokinetic data obtained [39].

## 4. Materials and Methods

### 4.1. Materials

Curcumin was purchased from Sigma-Aldrich company (St. Louis, MO, USA), Atorvastatin calcium was kindly obtained as a gift from the Egyptian Group for Pharmaceutical Industries (EGPI) (El Obour, Egypt). Polyoxyl 40 hydrogenated castor oil (Kolliphor^®^ RH 40), Polyoxyl 15 hydroxystearate (Solutol^®^ HS 15) and 3-(4,5-dimethylthiazol-2-yl)-2,5 diphenyltetrazolium bromide (MTT) were purchased from Sigma-Aldrich company (Darmstadt, Germany). Soya bean lecithin (Epikuron^®^ 200) was kindly provided as a gift from Cargill Deutschland GmbH (Hamburg, Germany). Oleic acid was purchased from Chemajet Chemical Company (Alexandria, Egypt). Isopropyl myristate (96%) was purchased from ACROS Organics (Geel, Belgium). Sodium chloride and acetone were purchased from El Nasr Pharmaceutical Chemicals Co. (ADWIC) (Cairo, Egypt). Ethanol, acetonitrile (HPLC analytical grade) and dimethyl sulfoxide (DMSO) were purchased from Fisher Scientific Co. (Waltham, NJ, USA). Folic acid was purchased from Qualikems Fine Chem Pvt. Ltd. (Vadodara, India). SERVAPOR dialysis tubing, MWCO 12000-14000 RC, diameter 21 mm, was purchased from SERVA Electrophoresis GmbH (Heidelberg, Germany). Human glioblastoma multiforme cell line (U87) was purchased from the American Type Culture Collection (ATCC, Manassas, VA, USA). Uranyl acetate was purchased from Allied Signal (Hanover, Germany). Dulbecco’s Modified Eagle Medium (DMEM) high glucose, fetal bovine serum and penicillin/streptomycin mixture were purchased from Gibco, Thermo Fischer Scientific (Waltham, NJ, USA). Sodium dihydrogen phosphate was purchased from Al Nasr company (Cairo, Egypt). Chloramine- T (CAT), N-chloro-p-toluene sulphonamide sodium salt and sodium metabisulphite (Na_2_S_2_O_5_) were purchased from Sigma-Aldrich Chemie GmbH (Taufkirchen, Germany). Carrier free- sodium [^131^I] Iodide was obtained from a radioisotope production facility (Inshas, Egypt). Disodium hydrogen phosphate (Na_2_HPO_4_.2H_2_O), chloroform, trifluoroacetic acid (TFA), formic acid, ketamine HCl, xylazine and isoflurane were purchased from Merck KGaA, (Darmstadt, Germany). Iodogen (1,3,4,6-tetrachloro-3α,6α-diphenylglycouril) was purchased from Aldrich Chemical Company (Steinheim, Germany). Biomaterial Hemolytic Assay Kit (HaemoScan, Groningen, Netherlands).

### 4.2. Methods

#### 4.2.1. Preparation of At-Cu LNCs

In this study, a binary miscible oil system was used to optimize drugs’ solubility. Atorvastatin was preferentially solubilized in oleic acid, whereas curcumin demonstrated enhanced solubility in isopropyl myristate, consistent with previously reported findings [89,90]; therefore, the selected oil system was oleic acid and isopropyl myristate at a ratio of 1:1. The formulation incorporated soybean lecithin (100 mg) as a lipophilic surfactant and solutol HS15 as a nonionic surfactant [91]. PIT method was selected for the preparation of At-Cu LNCs. Utilizing the quantities specified in Table 1, a mixture of the drugs, oils, surfactants, and 2.5 mL of distilled water containing 100 mg sodium chloride was subjected to three thermal cycling intervals, alternating between 85 °C and 55 °C, within a sealed glass vial under continuous magnetic stirring. Subsequently, ice-cold water (4 °C) was added to reach a final formulation weight of 10 g, followed by a 10 min stirring period [91,92]. Using a Box–Behnken design, concentration ranges were chosen based on literature reports and initial solubility tests to examine the effects of oil concentration, solutol HS15 concentration, and total drug load on particle size [23,24].

#### 4.2.2. Determination of PS, PDI and ZP of At-Cu LNCs

Samples were diluted 100-fold with deionized water and analyzed by dynamic light scattering (Zetasizer Nano ZS, Malvern Instruments, Malvern, UK). PS and PDI were measured using a disposable cuvette, ZP was determined using the reusable capillary zeta cell.

#### 4.2.3. Preparation of FA-At-Cu LNCs

**Figure 10:**
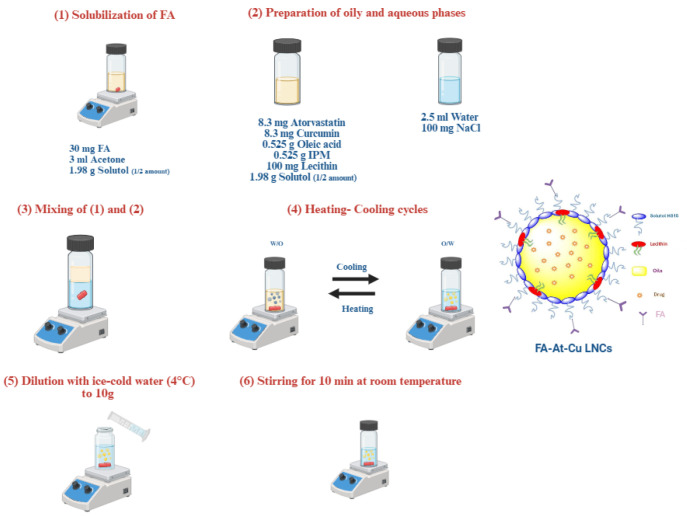
Schematic illustration for the method of preparation of FA-At-Cu LNCs.

The optimized formulation, composed of 39.60% *w*/*w* solutol, 10.50% *w*/*w* oils and 16.60 mg total drugs, was selected using Design Expert^®^ software based on achieving the smallest PS, then prepared and experimentally evaluated to verify agreement between predicted and actual values. For surface functionalization, the optimal concentration of FA was determined through a solubility study in acetone, which showed complete solubility up to 30 mg. Visible sedimentation was observed at 40 mg, indicating limited solubility at higher loading. Accordingly, LNCs functionalized with 10, 20, and 30 mg FA were prepared and assessed for PS, PDI, and ZP. As illustrated in Figure 10, FA was dissolved in acetone with half the amount of solutol, then incorporated into the oily phase (composed of the other half of solutol, 10.50%w/w oils and 16.60 mg total drugs). This oily phase was then mixed with the aqueous phase (composed of 100 mg sodium chloride in 2.5 mL distilled water). Then the mixture was subjected to three thermal cycling intervals (85 °C and 55 °C) under magnetic stirring. Finally ice-cold water was added till 10 g formulation was obtained. Stirring then continued for 10 min at room temperature. Acetone was removed during the heating cycles by occasional vial opening at 55 °C, as previously reported [93,94]. To delineate whether the interaction between FA and LNCs was physical or chemical in nature, Fourier transform infrared (FTIR) analysis of At-Cu LNCs and FA-At-Cu LNCs was conducted (RAM II FT-Raman, Bruker^®^, Billerica, MA, USA).

#### 4.2.4. XPS for Optimized At-Cu LNCs and FA-At-Cu LNCs

XPS is a surface-sensitive and quantitative technique that analyzes the elemental composition and chemical states of a material by irradiating its surface with an X-ray beam and measuring the emitted photoelectrons. It calculates the electrons and kinetic energy that escape from atoms on a material’s surface determining its elemental composition [95]. This technique was used to confirm the conjugation of FA to the surface of the selected LNCs using the unconjugated system as a control. Aliquots of At-Cu LNCs and FA-At-Cu LNCs were carefully deposited onto XPS-compatible silicon wafer substrates and vacuum-dried under controlled conditions until thin, uniform films were formed. These solid-state films were subsequently subjected to XPS analysis (XPS, K-Alpha, Thermo Scientific, Waltham, MA, USA).

#### 4.2.5. Morphology by TEM for Optimized At-Cu LNCs and FA-At-Cu LNCs

TEM was used to visualize the morphological characteristics of the optimized LNCs and their functionalized counterpart as previously described [4,96]. A drop of diluted nanocapsule dispersion (1:1000 with deionized water) was placed onto carbon-coated copper grids, stained with uranyl acetate solution (2% *w*/*v*) as a negative stain for 4 min and then air-dried at room temperature after removal of excess liquid with filter paper, followed by visualization (TEM, JEM-1400, JEOL Ltd., Tokyo, Japan).

#### 4.2.6. In Vitro Drug Release Evaluation

In vitro release of atorvastatin and curcumin from optimized At-Cu LNC and FA-At-Cu LNC formulations was performed by using dialysis bag method [47]. Before conducting the release study, sink conditions were ensured for both drugs in the release medium, which consisted of deionized water with a pH of 7.4 and 4% Kolliphor RH 40. One milliliter of the formulation, containing 830 µg of each drug, was placed in a cellulose dialysis bag and immersed in 10 mL of release medium within a stoppered vial. The vials were then placed in a thermostatic shaker adjusted at 37 °C and 100 rpm [91]. The experiment was conducted in triplicate. A sample of one milliliter was then withdrawn and replenished with fresh media at the following time intervals: 30 min, 1–8 and 24 h. The withdrawn samples were analyzed using high-performance liquid chromatography (HPLC, Shimadzu Corporation, Kyoto, Japan) via a low-pressure gradient pump mode. The mobile phase initially consisted of 60% acetonitrile and 40% water, with a gradual increase in acetonitrile concentration to 80% over the first 10 min, followed by a reduction back to 60% over the subsequent 3 min. The system utilized was a Nexera LC-30AD (Shimadzu corporation, Kyoto, Japan) equipped with a SIL-30AC autosampler and a photodiode array detector (LC-2030/2040), along with a Kromasil 100-5-C18 column (4.6 × 250 mm, Kromasil, Bohus, Sweden). Each chromatographic run was conducted over a 13 min period with a sampling speed of 5 µL/s and a flow rate of 0.7 mL/min. Detection wavelengths were set at 246 nm for atorvastatin calcium and 420 nm for curcumin, with all analyses performed at a controlled temperature of 25 ± 2 °C. The method was validated according to standard guidelines in our laboratory in terms of linearity, sensitivity and accuracy [97,98]. Worthy to note that release was calculated as a percentage of the total drug initially added based on the well-known high loading capacity of LNCs reaching almost 100%, coinciding with several reports [44,57,91,99].

#### 4.2.7. Studying the Effect of Storage on the Physical Stability of the Optimized LNCs

For 3 months, At-Cu LNCs and FA-At-Cu LNCs were stored at 4 ± 1 °C in tightly sealed vials in the refrigerator. Samples were then analyzed for PS, PDI and ZP to assess their stability [4].

#### 4.2.8. MTT Assay for Cell Viability

U87 cells were maintained in DMEM supplemented with 10% fetal bovine serum and antibiotics (100 U/mL penicillin, 100 µg/mL streptomycin) at 37 °C in a humidified 5% CO_2_ atmosphere under sterile conditions [100]. To ensure accurate experimental comparisons, all experiments included two control groups: a negative control of cells incubated in drug-free medium, and a solvent control with cells exposed to drug-free medium containing 0.5% DMSO, to evaluate any potential solvent toxicity [30]. Cell viability was determined using the MTT assay after a 24 h incubation of U87 cells with various drug formulations. These included free atorvastatin, free curcumin, a solution combining atorvastatin and curcumin, At-Cu LNCs and FA-At-Cu LNCs. Stock solutions (2 mg/mL) for individual free drugs and their combination were prepared in phosphate-buffered saline (PBS) containing 1% sterile DMSO to ensure complete solubilization, from which serial dilutions (25–800 µg/mL) were then made. At-Cu LNCs and FA-At-Cu LNCs were sonicated for 5 min prior to preparing their serial dilutions (0.025–250 µg/mL). Following incubation, the drug-containing medium was aspirated, and cells were re-incubated for 4 h at 37 °C with MTT-containing complete medium. The resultant formazan crystals were dissolved in acidified isopropanol, and absorbance was measured at 560 nm using PHERAstar microplate reader (BMG Labtech, Ortenberg, Germany). Cell viability was expressed as a percentage of untreated control cells, and half-maximal inhibitory concentrations (IC_50_) were calculated. Each concentration was tested in triplicate wells, and the entire experiment was repeated at least three times [101].

#### 4.2.9. In Vivo Pharmacokinetic Evaluation of Radiolabeled Drug-Loaded Nanocapsules in Glioma-Bearing Mice

Atorvastatin was radiolabeled with iodine-131 using the iodogen-coated tube method, while curcumin was labeled via electrophilic substitution employing chloramine-T as the oxidizing agent [102,103,104]. Labeling conditions were systematically optimized by varying pH, drug concentration, oxidant amount, and reaction time. Radiochemical purity and identity were assessed using ITLC-SG (Instant thin layer chromatography strips Silica gel, 5 × 20 cm, Gelman Instrument Company, Ann Arbor, MI, USA), RP-HPLC, and electrophoresis, confirming efficient incorporation of carrier-free ^131^I. Purification was achieved by RP-HPLC to remove free iodine and impurities. The resulting [^131^I]-atorvastatin and [^131^I]-curcumin formulations demonstrated high radiochemical yield and purity, suitable for subsequent in vivo biodistribution studies. The radiochemical yield was determined by calculating the percentage of the total radioactivity attributed to the radio-iodinated compound, using the following equation [105,106]:

$$Radiochemical\;yield\;\left(\%\right)=\frac{Activity\;of\;labeled\;compond}{Total\;activity\;}\;\times 100\;\;\;\;\;\;$$

The stability of the radiolabeled compounds was assessed under two sets of in vitro conditions. At room temperature, to evaluate physicochemical stability, the radiolabeled compound was stored at room temperature (25 °C) for up to 72 h. Aliquots were withdrawn at predetermined time points (1, 6, 12, 24, 48, and 72 h) and analyzed for radiochemical purity using the previously described chromatographic methods. Stability in serum was assessed by incubating the radiolabeled compound in freshly prepared serum at a compound-to-serum volume ratio of 1:8. The mixture was maintained at 37 °C to simulate physiological conditions. At the same time intervals (1, 6, 12, 24, 48, and 72 h post-incubation), 2 mL samples were collected in triplicate. The radiochemical integrity of each sample was then evaluated to detect any potential deiodination or degradation over time.

All animal procedures were conducted in accordance with arrive guidelines and were approved by the Research Ethics Committee at the Faculty of Pharmacy, The British university in Egypt, with approval number (EX-2508) [107]. Male BALB/c mice (6 weeks old, weighing 20–25 g) were utilized for the study. Prior to the start of experiments, animals were acclimatized for two weeks under controlled environmental conditions (25 ± 2 °C, 12 h light/dark cycle) in group-housed (3–5 mice/cage) polypropylene cages at the animal house of the Egyptian Atomic Energy Authority. They were fed with constant chow (46.4% carbohydrate, 23.6% protein, 3% fat and 27% calcium, phosphorous and fibers) and water ad libitum. For induction of brain tumor in mice, they were anesthetized through intraperitoneal injection of 0.1 mL of ketamine-xylazine solution containing ketamine HCl (25 mg/mL) and xylazine (2.5 mg/mL) as described in current protocols [108]. To implant cells intracranially, a calibrated 26-gauge 701N Hamilton micro-syringe (Micromeasure B.V., The Hague, The Netherlands) was used to inject 5 µL of 2.5 × 10^6^ cells of CT-2A glioma cell suspension intracranially through the post glenoid foramen as previously reported [109]. The right auricle was slowly pushed forward during the procedure, exposing the site of insertion. The syringe was held at an appropriate angle, and the cell suspension was slowly injected over 30 s. On completion, the needle was removed gently while applying light finger pressure to prevent bleeding. Additional pressure was applied for an additional 30 s after the removal of the needle to achieve hemostasis.

One hundred and eighty BALB/c mice were randomly assigned into six experimental groups, each further divided into six subgroups (n = 5 per subgroup), corresponding to specific time points post-injection: 0.5, 1, 2, 4, 8, and 24 h. All treatments were administered intravenously via the tail vein at a dose of 2 mg/kg for both atorvastatin and curcumin. Formulations were prepared under aseptic conditions using sterile water for injection and a laminar flow hood.

Group 1: LNCs containing [^131^I]-labeled atorvastatin and unlabeled curcumin.

Group 2: LNCs containing unlabeled atorvastatin and [^131^I]-labeled curcumin.

Group 3: FA-LNCs containing [^131^I]-labeled atorvastatin and unlabeled curcumin.

Group 4: FA-LNCs containing unlabeled atorvastatin and [^131^I]-labeled curcumin.

Group 5: Free [^131^I]-labeled atorvastatin solution (control).

Group 6: Free [^131^I]-labeled curcumin solution (control).

At each time point, five mice per group were anesthetized using isoflurane, and blood samples were collected by cardiac puncture into pre-heparinized polypropylene tubes. Mice were then euthanized by CO_2_ inhalation, and organs including the brain, liver, and kidneys and others were excised, rinsed with physiological saline, gently blotted, and weighed. Tissue and plasma samples were analyzed for radioactivity using a well-type γ-scintillation counter (SR-7,Nuclear Enterprises Ltd., San Carlos, CA, USA) and data were expressed as the percentage of the injected dose per gram of tissue (%ID/g) [110].

The mean radioactivity values (represented as % injected dose per gram of tissue, %ID/g) acquired at various time points were examined using PKSolver, a Microsoft Excel add-in, in order to evaluate the pharmacokinetic behavior and tissue distribution of the radiolabeled formulations. Key pharmacokinetic parameters, such as the mean residence time (MRT) and the area under the curve (AUC (0–24 h)) for each organ and formulation, were computed using the non-compartmental analysis (NCA) model. AUC values based on time-radioactivity data were calculated using the trapezoidal rule [111].

Two quantitative parameters were computed using the AUC values obtained from the biodistribution study in order to assess the targeting performance and tissue selectivity of the formulations. The following equation was used to calculate tumor targeting efficiency (%):

$$Tumor\;targeting\;efficiency\;\left(\%\right)=\;\frac{{AUC}_{0-24}\;glioma}{{AUC}_{0-24\;}\;blood}\;x100\;\;$$

This metric illustrates how the formulation preferentially accumulates in glioma tissue as opposed to systemic circulation. Furthermore, the tumor tissue specificity index was computed using the following equation to evaluate the selectivity of glioma accumulation over healthy brain tissue:

(1) $$Tumor\;tissue\;specificity\;index=\;\frac{{AUC}_{0-24}\;glioma}{{AUC}_{0-24\;}\;brain}\;\;\;$$

According to earlier descriptions in nanoparticle pharmacokinetic studies, these AUC-based ratios are in line with accepted techniques for evaluating the tissue-targeting behavior of nanocarriers and radiolabeled drug delivery systems [112,113].

Using the Biomaterial Hemolytic Assay Kit (HaemoScan, Groningen, The Netherlands), an in vitro hemolysis assay was conducted to evaluate safety. This test measures the amount of hemoglobin released when erythrocytes and the tested formulations come into direct contact to assess hemocompatibility. Following the manufacturer’s instructions, the assay was performed in triplicate. Hemolysis was computed as a percentage of the sample’s total hemoglobin. Blood compatibility was confirmed by the minimal hemolysis (<5%) shown by all formulations [86].

#### 4.2.10. Statistical Analysis

All data are expressed as mean ± standard deviation (n = 3). Statistical analysis was performed using GraphPad Prism^®^ v8.4.3 (GraphPad Software Inc., San Diego, CA, USA). Differences between groups were evaluated using one-way ANOVA, followed by Tukey’s post hoc test for multiple comparisons, or unpaired *t*-test, as appropriate. The difference between means was considered statistically significant at *p* ≤ 0.05. Additionally, Design-Expert^®^ v13.0.0 (Stat-Ease Inc., Minneapolis, MN, USA) was employed for experimental design and optimization studies.

## 5. Conclusions

This work demonstrates that folic acid-functionalized lipidic nanocapsules (FA-LNCs) co-encapsulating curcumin and atorvastatin have good physicochemical characteristics needed for systemic stability and efficient blood–brain barrier penetration. Nanoencapsulation significantly altered the pharmacokinetics and biodistribution of both agents, improving glioma targeting with high tumor specificity indices, decreasing hepatic clearance, and extending systemic circulation, according to the radiolabeled in vivo studies. These findings point to FA-LNCs as a potentially effective platform that can greatly enhance the therapeutic index of curcumin and atorvastatin in glioma treatment. Beyond improved delivery, the combination of curcumin and atorvastatin is expected to exert synergistic effects in vivo through their complementary mechanisms, further enhancing the therapeutic potential of FA-LNCs; therefore, future pharmacodynamic studies would be valuable in this regard. More significantly, this study provides a proof of concept that rationally designed ligand-targeted nanocarriers can transform current medications into effective, brain-directed anticancer treatments, establishing the foundation for translational progress toward clinical use.

## Figures and Tables

**Table 1:** At-Cu LNCs composition, particle size (PS), polydispersity index (PDI) and zeta potential (ZP) values generated by Box–Behnken design.

Formulation Code *	Solutol Conc %*w*/*w*	Oils Conc %*w*/*w*	Amount of Drug (mg)	PS (nm) Mean ± SD	PDI Mean ± SD	ZP (mV) Mean ± SD
At-Cu LNCs1	10	10	22.5	162.30 ± 12.16	0.44 ± 0.08	−26.00 ± 1.41
At-Cu LNCs2	40	10	22.5	130.15 ± 0.63	0.34 ± 0.09	−14.25 ± 0.35
At-Cu LNCs3	10	25	22.5	238.65 ± 8.55	0.35 ± 0.13	−22.65 ± 4.45
At-Cu LNCs4	40	25	22.5	160.50 ± 14.70	0.42 ± 0.05	−11.36 ± 2.60
At-Cu LNCs5	10	17.5	15	170.50 ± 7.07	0.33 ± 0.05	−26.55 ± 6.01
At-Cu LNCs6	40	17.5	15	124.40 ± 12.44	0.32 ± 0.09	−11.45 ± 1.34
At-Cu LNCs7	10	17.5	30	186.45 ± 11.66	0.39 ± 0.08	−26.00 ± 0.98
At-Cu LNCs8	40	17.5	30	138.20 ± 3.81	0.35 ± 0.01	−13.90 ± 1.97
At-Cu LNCs9	25	10	15	120.75 ± 14.07	0.32 ± 0.05	−17.55 ± 1.90
At-Cu LNCs10	25	25	15	152.35 ± 15.76	0.44 ± 0.05	−17.30 ± 0.56
At-Cu LNCs11	25	10	30	159.55 ± 8.55	0.46 ± 0.11	−21.10 ± 1.97
At-Cu LNCs12	25	25	30	172.25 ± 15.76	0.45 ± 0.07	−15.85 ± 0.07
At-Cu LNCs13	25	17.5	22.5	264.25 ± 10.96	0.45 ± 0.18	−19.50 ± 2.68
At-Cu LNCs13(1)	25	17.5	22.5	269.95 ± 1.90	0.38 ± 0.03	−19.30 ± 0.57
At-Cu LNCs13(2)	25	17.5	22.5	282.55 ± 8.27	0.37 ± 0.00	−17.70 ± 2.96

* At-Cu LNCs13(1) and 13(2) are replicates of formulation At-Cu LNCs13 (center point formulation), intended for assessing the experimental error and ensuring the statistical validity of the Box–Behnken design.

**Table 2:** ANOVA results for the quadratic model evaluating the effects of formulation variables on the particle size of At-Cu LNCs, as generated by Design Expert^®^ software.

Source	Sum of Squares	df	Mean Square	F-Value	*p*-Value	
Model	41177.37	9	4575.26	28.64	0.0009	significant
A-Solutol conc	5235.20	1	5235.20	32.77	0.0023	
B-oils conc	2850.12	1	2850.12	17.84	0.0083	
C-Amount of drugs	977.93	1	977.93	6.12	0.0562	
AB	529.00	1	529.00	3.31	0.1284	
AC	1.16	1	1.16	0.0072	0.9355	
BC	89.30	1	89.30	0.5591	0.4883	
A^2^	8451.78	1	8451.78	52.91	0.0008	
B^2^	9795.30	1	9795.30	61.32	0.0005	
C^2^	17,844.39	1	17844.39	111.71	0.0001	
Residual	798.66	5	159.73			
Lack of Fit	623.28	3	207.76	2.37	0.3106	not significant
Pure Error	175.38	2	87.69			
Cor Total	41,976.03	14				

**Table 3:** Composition of the optimized formulation as suggested by Design-Expert^®^ software, along with predicted and actual responses.

	Solutol Conc %*w*/*w*	Oils Conc %*w*/*w*	Amount of Drug (mg)	Mean Particle Size (nm) ± SD
Predicted	39.60	10.50	16.60	95.68
Actual				97.98 ± 2.27

**Table 4:** Effect of increasing folic acid Concentration on the particle size, polydispersity index, and zeta potential of. FA-At-Cu LNCs.

Amount of Folic Acid (mg)	Particle Size (nm) Mean ± SD	Polydispersity Index Mean ± SD	Zeta Potential (mV) Mean ± SD
10	130.70 ± 2.27	0.30 ± 0.03	−12.80 ± 0.42
20	172.65 ± 1.76	0.50 ± 0.01	−11.45 ± 0.35
30	181.60 ± 2.83	0.40 ± 0.02	−11.90 ± 2.80

**Table 5:** Effect of three months storage at 4 ± 1 °C on the particle size, polydispersity index and zeta potential on the selected At-Cu LNCs and FA-At-Cu LNCs.

	At-Cu LNCs		FA-At-Cu LNCs	
	Before Storage	After 3 Months	Before Storage	After 3 Months
Particle size (nm)	97.98 ± 2.27	106.70 ± 2.30	181.60 ± 2.83	184.90 ± 1.90
Polydispersity	0.32 ± 0.07	0.30 ± 0.00	0.40 ± 0.02	0.46 ± 0.05
Zeta potential (mV)	−15.85 ± 1.35	−16.31 ± 2.30	−11.90 ± 2.80	−12.00 ± 3.20

**Table 6:** Pharmacokinetic parameters of atorvastatin & curcumin following intravenous administration of different formulations in glioma-bearing mice.

	Atorvastatin			Curcumin		
	Free Solution	LNCs	FA LNCs	Free Solution	LNCs	FA LNCs
MRT in blood	9.77 ± 2.57	30.04 ± 0.00	30.61 ± 2.42	12.08 ± 0.02	26.13 ± 3.35	26.17 ± 1.59
AUC (0–24 h) Blood	71.47 ± 17.13	151.08 ± 25.64	163.08 ± 31.07	72.27 ± 15.44	236.76 ± 52.04	217.51 ± 49.21
AUC (0–24 h) Brain	0.99 ± 0.36	3.50 ± 1.30	4.97 ± 1.77	1.47 ± 0.44	6.97 ± 2.35	6.68 ± 2.26
AUC (0–24 h) Glioma	3.52 ± 1.13	14.51 ± 5.13	28.70 ± 10.09	3.72 ± 1.34	17.50 ± 6.15	46.20 ± 15.77
Tumor targeting efficiency %	4.88 ± 0.40	9.45 ± 1.79	17.32 ± 2.89	5.07 ± 0.77	7.28 ± 0.99	20.95 ± 2.51
Tumor tissue specificity index	3.57 ± 0.16	4.15 ± 0.08	5.77 ± 0.02	2.53 ± 2.47	2.51 ± 0.04	6.91 ± 0.01

MRT represented in (h), AUC represented in (%ID*h/g tissue). Values represent pharmacokinetic parameters calculated from radioactivity–time profiles obtained as mean ± SD from five independent animals per time interval).

**Table 7:** Area under the curve (AUC (0–24 h)) values of atorvastatin and curcumin in clearance-associated organs following intravenous administration of different formulations.

	Atorvastatin			Curcumin		
	Free Solution	LNCs	FA LNCs	Free Solution	LNCs	FA LNCs
AUC (0–24 h) liver	440.88 ± 49.33	267.40 ± 55.87	220.50 ± 37.42	711.50 ± 114.83	658.50 ± 117.80	660.50 ± 117.38
AUC (0–24 h) Spleen	3.90 ± 0.90	57.85 ± 9.82	38.05 ± 6.48	7.07 ± 1.70	114.37 ± 20.18	102.37 ± 17.87
AUC (0–24 h) kidneys	75.39 ± 14.24	50.98 ± 8.84	41.95 ± 7.16	57.87 ± 12.47	29.50 ± 7.21	29.50 ± 6.53
AUC (0–24 h) Intestine	398.22 ± 52.90	197.50 ± 41.12	174.87 ± 29.67	526.50 ± 111.63	195.97 ± 40.55	201.77 ± 34.55

AUC represented in (%ID*h/g tissue).

**Figure A1:**
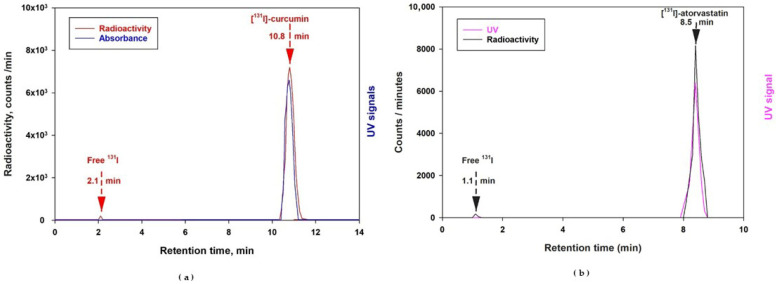
HPLC radio chromatogram of: (**a**) purified [^131^I]-atorvastatin compound, (**b**) purified [^131^I]-Curcumin compound.

## Data Availability

The datasets for this work are available from the authors upon request.

## Supplementary Materials

The following supporting information can be downloaded at: https://www.mdpi.com/article/10.3390/ph18111623/s1, Figure S1. FTIR spectrum of: (a) At-Cu LNCs, (b) FA-At-Cu LNCs, with evidence of successful folic acid chemical conjugation demonstrated by complete disappearance of the characteristic free carboxylic acid peak of FA at 2500–3500 cm^−1^ in the FA-At-Cu LNCs spectrum, indicating its participation in covalent bond formation, as well as the decreased broad O–H stretching band of Solutol at 3371.71 cm^−1^ in the FTIR spectrum of FA-At-Cu LNCs, suggesting involvement in ester bond formation with FA.

## Abbreviations

The following abbreviations are used in this manuscript:

LNCs	Lipidic nanocapsules
FA-LNCs	Folic acid functionalized lipidic nanocapsules
PIT	Phase inversion temperature
PS	Particle size
PDI	Polydispersity index
ZP	Zeta potential
XPS	X-ray photoelectron spectroscopy
TEM	Transmission electron microscopy
HPLC	High performance liquid chromatography
MRT	Mean residence time
AUC	Area under the curve
PEG	Polyethylene glycol
DMSO	Dimethyl sulfoxide
DMEM	Dulbecco’s Modified Eagle Medium
EPR	Enhanced permeability and retention
MPS	Mononuclear phagocyte system
At-Cu LNCs	Atorvastatin and curcumin coloaded lipidic nanocapsules
FA-At-Cu LNCs	Folic acid functionalized atorvastatin and curcumin coloaded lipidic nanocapsules

## Appendix B

**Table A1:** Time-dependent biodistribution of atorvastatin (%ID/g) in selected organs following intravenous administration of different formulations in glioma-bearing mice.

Organ	Formulation	0.5 h	1 h	2 h	4 h	8 h	24 h
Heart	Solution	1.50± 0.30	1.30 ± 0.27	1.20 ± 0.22	1.10 ± 0.18	0.90 ± 0.15	0.20 ± 0.01
	LNC	1.30 ± 0.21	1.10 ± 0.13	1.00 ± 0.18	1.00 ± 0.12	0.80 ± 0.12	0.15 ± 0.02
	FA-LNCs	1.20 ± 0.14	1.00 ± 0.12	0.90 ± 0.11	0.80 ± 0.10	0.60 ± 0.07	0.15 ± 0.02
Lungs	Solution	0.60 ± 0.09	1.20 ± 0.05	1.10 ± 0.06	0.80 ± 0.05	0.50 ± 0.08	0.18 ± 0.01
	LNC	0.70 ± 0.08	1.30 ± 0.21	1.20 ± 0.14	0.90 ± 0.15	0.60 ± 0.11	0.25 ± 0.04
	FA-LNCs	0.80 ± 0.10	1.40 ± 0.17	1.20 ± 0.14	1.00 ± 0.12	0.70 ± 0.08	0.30 ± 0.04
Stomach	Solution	6.00 ± 0.45	8.15 ± 1.27	10.04 ± 1.37	13.43 ± 2.1	15.20 ± 1.70	4.00 ± 0.15
	LNC	5.00 ± 0.66	6.80 ± 0.84	8.00 ± 1.13	9.50 ± 0.97	7.50 ± 0.77	2.30 ± 0.23
	FA-LNCs	4.50 ± 0.54	6.00 ± 0.72	7.00 ± 0.84	9.00 ± 1.08	6.00 ± 0.72	2.00 ± 0.24
Bone	Solution	0.80 ± 0.09	1.10 ± 0.12	1.00 ± 0.15	0.90 ± 0.14	0.68 ± 0.10	0.40 ± 0.06
	LNC	0.80 ± 0.10	1.10 ± 0.12	1.00 ± 0.19	0.90 ± 0.17	0.68 ± 0.10	0.40 ± 0.08
	FA-LNCs	0.80 ± 0.10	1.00 ± 0.12	1.00 ± 0.12	0.90 ± 0.11	0.70 ± 0.08	0.40 ± 0.05
Muscle	Solution	1.50 ± 0.22	1.30 ± 0.20	1.10 ± 0.17	0.80 ± 0.12	0.40 ± 0.10	0.20 ± 0.01
	LNC	1.50 ± 0.22	1.30 ± 0.24	1.10 ± 0.18	0.80 ± 0.09	0.40 ± 0.05	0.20 ± 0.04
	FA-LNCs	1.50 ± 0.18	1.30 ± 0.16	1.10 ± 0.13	0.80 ± 0.10	0.40 ± 0.05	0.20 ± 0.02
Urine	Solution	2.40 ± 0.12	3.54 ± 0.11	5.58 ± 0.62	5.71 ± 1.30	7.21 ± 1.10	8.20 ± 1.30
	LNC	2.00 ± 0.37	3.00 ± 0.43	4.50 ± 0.89	4.50 ± 0.88	5.50 ± 0.84	4.50 ± 0.75
	FA-LNCs	1.80 ± 0.22	2.50 ± 0.30	3.50 ± 0.42	4.00 ± 0.48	4.50 ± 0.54	3.50 ± 0.42
Thyroid glands	Solution	0.05 ± 0.01	0.07 ± 0.01	0.18 ± 0.02	0.22 ± 0.02	0.03 ± 0.01	0.03 ± 0.0
	LNC	0.03 ± 0.00	0.03 ± 0.00	0.02 ± 0.00	0.02 ± 0.00	0.02 ± 0.00	0.01 ± 0.00
	FA-LNCs	0.03 ± 0.00	0.03 ± 0.00	0.02 ± 0.00	0.02 ± 0.00	0.02 ± 0.00	0.01 ± 0.00

**Table A2:** Time-dependent biodistribution of curcumin (%ID/g) in selected organs following intravenous administration of different formulations in glioma-bearing mice.

Organ	Formulation	0.5 h	1 h	2 h	4 h	8 h	24 h
Heart	Solution	2.00 ± 0.30	1.80 ± 0.27	1.60 ± 0.24	1.30 ± 0.20	1.20 ± 0.18	1.10 ± 0.17
	LNC	2.30 ± 0.30	2.10 ± 0.30	1.90 ± 0.30	1.60 ± 0.20	1.30 ± 0.20	1.00 ± 0.20
	FA-LNCs	2.20 ± 0.30	2.00 ± 0.30	1.80 ± 0.30	1.50 ± 0.25	1.20 ± 0.20	1.00 ± 0.20
Lungs	Solution	3.00 ± 0.20	2.00 ± 0.10	2.00 ± 0.04	1.40 ± 0.36	1.00 ± 0.30	0.80 ± 0.05
	LNC	3.50 ± 0.40	3.00 ± 0.40	2.50 ± 0.30	2.00 ± 0.30	1.50 ± 0.20	1.00 ± 0.20
	FA-LNCs	3.40 ± 0.40	2.90 ± 0.35	2.40± 0.30	1.90 ± 0.25	1.40± 0.20	1.00 ± 0.20
Stomach	Solution	4.00 ± 0.60	4.30 ± 0.65	3.50 ± 0.68	4.00 ± 0.69	3.20 ± 0.63	3.10 ± 0.09
	LNC	3.80 ± 0.50	3.50 ± 0.50	3.30 ± 0.40	3.10 ± 0.40	2.90 ± 0.30	2.70 ± 0.30
	FA-LNCs	3.70 ± 0.50	3.40 ± 0.50	3.20 ± 0.40	3.00 ± 0.40	2.80 ± 0.35	2.60 ± 0.30
Bone	Solution	1.00 ± 0.15	0.90 ± 0.14	0.80 ± 0.12	0.70 ± 0.11	0.60 ± 0.09	0.50 ± 0.08
	LNC	0.80 ± 0.10	0.70 ± 0.10	0.60 ± 0.10	0.60 ± 0.10	0.50 ± 0.10	0.40 ± 0.10
	FA-LNCs	0.80 ± 0.10	0.70 ± 0.10	0.60 ± 0.10	0.60 ± 0.10	0.50 ± 0.10	0.40 ± 0.05
Muscle	Solution	1.60 ± 0.24	1.40 ± 0.21	1.30 ± 0.20	1.20 ± 0.18	1.10 ± 0.17	1.00 ± 0.15
	LNC	1.90 ± 0.20	1.70 ± 0.20	1.50 ± 0.20	1.30 ± 0.20	1.20 ± 0.20	1.00 ± 0.10
	FA-LNCs	1.80 ± 0.20	1.60 ± 0.20	1.40 ± 0.20	1.30 ± 0.20	1.20 ± 0.15	1.00 ± 0.15
Urine	Solution	4.50 ± 0.68	5.20 ± 0.78	5.60 ± 0.84	5.40 ± 0.81	4.60 ± 0.69	3.90 ± 0.58
	LNC	1.50 ± 0.20	1.60 ± 0.20	1.60 ± 0.20	1.40 ± 0.20	1.20 ± 0.20	1.00 ± 0.20
	FA-LNCs	1.50 ± 0.20	1.60 ± 0.20	1.60 ± 0.20	1.40 ± 0.20	1.20 ± 0.20	1.00 ± 0.15
Thyroid glands	Solution	0.06 ± 0.01	0.04 ± 0.00	0.04 ± 0.00	0.03 ± 0.01	0.02 ± 0.00	0.01 ± 0.00
	LNC	0.50 ± 0.08	0.40 ± 0.06	0.40 ± 0.06	0.30 ± 0.05	0.20 ± 0.03	0.20 ± 0.03
	FA-LNCs	0.45 ± 0.07	0.35 ± 0.05	0.35 ± 0.05	0.25 ± 0.04	0.15 ± 0.02	0.15 ± 0.02
