# Supplementary material for: Folic Acid-Decorated Lipidic Nanocapsules Co-Loaded with Atorvastatin and Curcumin to Enhance Glioma Targeting in Mice
Source: Pharmaceuticals (Basel). 2025 Oct 27;18(11):1623. doi: 10.3390/ph18111623 (PMC12655235; doi:10.3390/ph18111623)
Supplement: Supplementary file 1 [file pharmaceuticals-18-01623-s001.zip › pharmaceuticals-3926258-supplementary.pdf]

<u>**Supplementary file 1**</u>

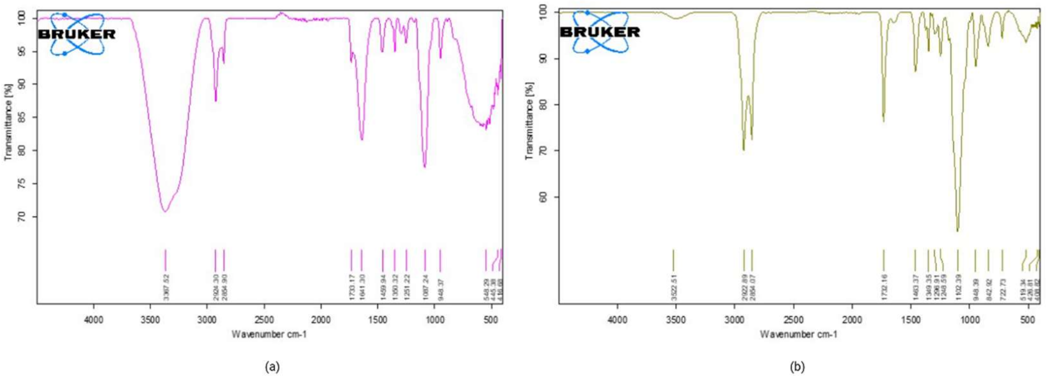

Figure S1. FTIR spectrum of: (a) At-Cu LNCs, (b) FA-At-Cu LNCs, with evidence of successful folic acid chemical conjugation demonstrated by complete disappearance of the characteristic free carboxylic acid peak of FA at 2500-3500 cm^{-1} in the FA-At-Cu LNCs spectrum, indicating its participation in covalent bond formation, as well as the decreased broad O–H stretching band of Solutol at 3371.71 cm^{-1} in the FTIR spectrum of FA-At-Cu LNCs, suggesting involvement in ester bond formation with FA.
